# Psychopathological Profiles of Adolescent Suicide Attempters: A Comprehensive Analysis

**DOI:** 10.1192/j.eurpsy.2025.446

**Published:** 2025-08-26

**Authors:** A. Garcia Fernandez, C. Martínez-Cao, I. Pérez-Díez, J. Andreo-Jover, W. Ayad-Ahmed, A. I. Cebriá, A. González-Pinto, I. Grande, M. Ruiz Veguilla, P. A. Sáiz

**Affiliations:** 1 Universidad de Oviedo, Oviedo; 2 Universidad Autónoma; 3 Hospital Universitario La Paz; 4 Universidad Complutense, Madrid; 5 Hospital Parc Taulí, Sabadell; 6 Hospital Universitario Araba, Vitoria; 7 Hospital Clinic, Barcelona; 8 Hospital Virgen del Rocio, Sevilla, Spain

## Abstract

**Introduction:**

Despite the identification of several risk factors, an understanding of the role of specific psychopathological profiles in predicting adolescent suicidal behaviours remains a key challenge in public health research.

**Objectives:**

The current study aimed to identify psychopathological profiles in suicidal adolescents and to analyse their association with suicide-related outcomes.

**Methods:**

A total of 285 adolescents aged 12 to 17 years [mean age (SD)=14.98 (1.51); females: 249 (87.40%)] were recruited from different hospitals in Spain. Latent profile analysis was performed to classify subgroups with similar patterns based on self-report Strengths and Difficulties Questionnaire. Logistic regression and generalised linear modelling were applied to examine the relationship between profile membership and suicidal behaviours.

**Results:**

Three psychopathological profiles were identified: internalizing symptom profile (52.63%), externalizing symptom profile (24.21%), and low symptom profile (25.58%). The predominantly female internalizing symptom profile members were more likely to report higher levels of psychopathological symptoms, including number of psychiatric diagnoses, depressive symptoms, and trauma (except sexual abuse). Additionally, they had more non-suicidal self-injury (NSSI) and suicidal thoughts and behaviours. Likewise, greater ideation intensity was associated with the internalizing symptom profile compared to other groups, while greater number of previous suicide attempts correlated with an increase in suicidal behaviours. Finally, higher levels of motor impulsivity were associated with a lower probability of suicidal behaviours.

**Conclusions:**

Identifying symptom profiles among adolescents who have attempted suicide allows us to establish reliable predictors for suicide prevention as well as personalised interventions, indicating the domains where these interventions are needed.

**Disclosure of Interest:**

None Declared

